# 68 6200-6700 steps per day are associated with better health and wellbeing among working-aged adults a

**DOI:** 10.1093/eurpub/ckae114.032

**Published:** 2024-09-26

**Authors:** Pauliina Husu, Henri Vähä-Ypyä, Kari Tokola, Harri Sievänen, Tommi Vasankari

**Affiliations:** The UKK Institute for Health Promotion Research, Tampere, Finland; The UKK Institute for Health Promotion Research, Tampere, Finland; The UKK Institute for Health Promotion Research, Tampere, Finland; The UKK Institute for Health Promotion Research, Tampere, Finland; The UKK Institute for Health Promotion Research, Tampere, Finland; Faculty of Medicine and Health Technology, Tampere University, Tampere, Finland

## Abstract

**Purpose:**

Physical activity (PA) is important for health and wellbeing, and the number of steps is one indicator of ambulatory PA. Guidelines of PA needed for health and wellbeing exist, but the adequate number of daily steps is equivocal. This study aimed at identifying potential step cut-points for selected indicators of health and wellbeing.

**Methods:**

The study is based on combined data from two cross-sectional population-based FinFit studies that measured physical behavior, including the daily number of steps, by a triaxial hip-worn accelerometer (UKK RM42) among 20-69-year-old adults. Receiver operator characteristics (ROC) curve analysis was used to analyze potential cut-points for steps in relation to measured waist circumference and blood lipids, as well as perceived health status, and physical and mental quality of life. The optimal cut-point was defined as the point closest to (0,1)-corner in the ROC plane.

**Results:**

Participants (n = 4302, mean age 50.4(SD 13.2) years, 62% women) used the accelerometer for at least 4 days, 24h/day, and took on average 7308 steps per day. The mean waist circumference was 97 cm in males and 88 cm in females. Mean values for total, LDL, and HDL cholesterol and triglycerides were 5.1, 3.1, 1.7, and 1.2 mmol/l, respectively. Most (87%) of the participants perceived their health status as at least good, and 51% reported their physical and mental quality of life to be on at least a median level.

Waist circumference, HDL cholesterol, triglycerides, perceived health status, and both physical and mental quality of life showed a statistically significant (p ≤ 0.001) association with the daily step number. The area under the curve values ranged from 0.53 (mental quality of life) to 0.65 (perceived health). The optimal step cut points ranged from 6123 steps for good perceived health status to 6618 steps for recommended HDL cholesterol.

**Conclusions:**

Working-aged adults should take at least 6200-6700 steps per day to have recommended waist circumference, HDL-cholesterol, and triglycerides, to perceive their health as good, and to have at least a medium level of quality of life.

**Funding source:**

the Finnish Ministry of Education and Culture

